# Feeding Management of African Rhinos (*Ceratotherium simum*, *Diceros bicornis*) in European Zoos

**DOI:** 10.1002/zoo.70031

**Published:** 2025-10-11

**Authors:** Gila Sauspeter, Marcus Clauss, Sylvia Ortmann, Andrew J. Abraham, Rebecca Biddle, Lars Versteege, Marcin Przybyło

**Affiliations:** ^1^ Clinic for Zoo Animals, Exotic Pets and Wildlife, Vetsuisse Faculty University of Zurich Zurich Switzerland; ^2^ Leipniz Institute for Zoo and Wildlife Reseach Berlin Germany; ^3^ Centre for Ecological Dynamics in a Novel Biosphere (ECONOVO), Section of EcoInformatics and Biodiversity, Department of Biology Aarhus University Aarhus Denmark; ^4^ Twycross Zoo Twycross UK; ^5^ Safaripark Beekse Bergen Hilvarenbeek the Netherlands; ^6^ Department of Animal Nutrition and Biotechnology, and Fisheries University of Agriculture in Krakow Krakow Poland

**Keywords:** health, husbandry, management, rhinoceros, roughage, salt

## Abstract

White rhinos (WR, *Ceratotherium simum*) are grazers and black rhinos (BR, *Diceros bicornis*) are browsers; this difference has been related to BR being more challenging in terms of husbandry. Previous reports indicated that BR in zoos often receive diets with a higher proportion of easily digestible compounds compared to other rhino species. Additionally, BR are susceptible to iron overload disorder (IOD). We compared the feeding methods and recommendations, and sampled diets and feces of 117 WR and 82 BR across 41 European zoos. The few available data for free‐ranging animals were also incorporated. The percentage of forage in the total estimated dry matter intake was 93% for WR and 77% for BR, matching fecal parameters indicating a higher diet digestibility in BR. BR received higher levels of (pelleted) compound feeds and produce than WR. Nevertheless, dietary fiber levels were similar to that reported for free‐ranging specimens in both species. Zoo diets were higher in several minerals (Na, P, Cu, Zn) than natural diets. However, zoo BR received diets with similar iron content to that described in free‐ranging BR diet items and fecal iron levels were not higher than those in the few samples of free‐ranging BR available, suggesting that iron levels in zoo BR diets are not overly excessive. Consequently, IOD appears to be more complex, associated with iron bioavailability and possibly non‐dietary causes. Grass hay was used in 100% of WR and 36% of BR (all BR facilities used lucerne hay). BR feces had levels of abrasive silica comparable to those of free‐ranging WR and much higher than free‐ranging BR, matching reports of excessive tooth wear in adult BR. Browse provision for BR could not be quantified but appeared low in general; no alternative forages (like sainfoin) were fed. Efforts to consistently provide high amounts of browse to BR must be increased and forage should be low in iron and abrasive silica.

## Graphical abstract

The average estimated diets (in % dry matter) of white rhinos (*C. simum*) and black rhinos (*D. bicornis*) in European zoos differ in the proportion of (pelleted) compound feed and other non‐forage items. The reason for this difference is elusive but possibly related to creating more complicated diets for browsers.

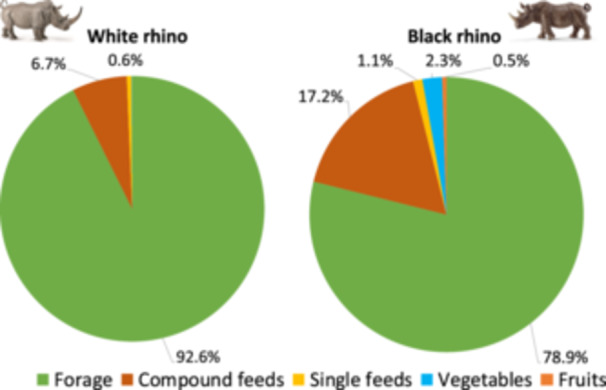

## 1 Introduction

African rhino species are up against multiple threats and diminishing numbers in the wild (Chanyandura et al. 2021). The white rhino (WR, *Ceratotherium simum*) is classified as near threatened and the black rhino (BR, *Diceros bicornis*) as critically endangered (IUCN 2024). Despite high poaching levels, in 2022 both African rhino populations were increasing for the first time in years (IUCN 2023). Likewise, populations of both species in European institutions have almost become self‐sustaining, but are still struggling with reaching the goal of population growth set by the European Association of Zoos and Aquaria's Ex‐situ Programme (EEP; Edwards et al. 2015; Versteege 2020). BR under human care may develop various health conditions (Dennis et al. 2007) and WR show limitations in their reproductive performance (Reid et al. 2012). Optimal husbandry is essential to maintain stable ex‐situ populations of WR and BR in Europe.

A recent study confirmed that zoo‐housed WR survive relatively longer than zoo‐housed BR, even though more significant improvements in survivorship have occured in BR (Wittwer et al. 2023). BR may have a greater tendency to stress and greater susceptibility to certain diseases or, as browsers versus grazers, they may be more difficult to maintain in managed care (Radeke‐Auer et al. 2023). Several diseases of historic importance in zoo‐housed BR may have decreased in prominence in recent years, yet iron overload disorder (IOD) is still detected to varying degrees in all adults at post ‐mortem examination (Radeke‐Auer et al. 2023). The etiopathology of IOD, its association with other metabolic syndromes (including reduced insulin sensitivity with obesity) and a general stress susceptibility, remain areas for investigation (Schook et al. 2015; Radeke‐Auer et al. 2023).

Nutrition is important in the maintenance of healthy zoo populations; it is considered key to preventing IOD in BR (Clauss et al. 2012; Sullivan et al. 2020; Duncan 2023). It has been difficult to directly link the increased incidence of health issues and the less successful husbandry of BR relative to other rhino species (Wittwer et al. 2023) in zoos to feeding protocols (Clauss and Hatt 2006; Duncan 2023). BR in zoos have higher levels of propionate (a certain volatile fatty acid) in their feces compared to other rhino species (Roth et al. 2019; Cersosimo et al. 2022), indicating the animals are fed a diet of more easily digestible carbohydrates (Clauss et al. 2005a; Grimm et al. 2017), typically not recommended for large herbivores. However, a current comparison of feeding practices of zoo‐housed rhino species is lacking.

The present study aimed to assess the current feeding practices for WR and BR in European facilities, and in particular to test whether BR receive diets with more easily digestible components, and whether distinct sources for dietary iron were present.

## 2 Materials and Methods

The first author visited 41 zoological institutions holding African rhinos in Europe between March 2022 and March 2023 (four zoos held both species.) The two species were assessed separately, so institutions were counted individually as WR holders and as BR holders and listed twice in the total number if they held both. A total of 117 WR and 82 BR were included (representing 95.3% of the European population at the time). WR institutions closer to the first author's location in Germany were visited preferentially.

A questionnaire guided data aquesition and interviews with zoo keepers. In addition, brief conversations with curators and veterinarians occurred. Institutions provided feeding and enclosure plans ahead of the visits, and medical records were made available. Weights were collected (WR: *n* = 22, 19% and BR: *n* = 33, 40%), or estimated by comparison to individuals of known weight. All feedings on the day of visit were observed. All feed items were noted and weighed when possible. Browse was not weighed, but number and sizes of branches were recorded. When it was not possible to attend every feeding, feed items were weighed ahead or a subset was used to estimate quantity and weight. All feed items except fresh food (produce and browse) were collected into paper bags. For hay, the samples represented grab samples from open bales.

An effort was made to obtain an individual fecal sample from each animal. If animals were permanently kept together in pairs or groups, a pooled fecal sample was taken. Samples were collected as freshly as possible and without contamination. Each pile of feces was photographed, the colour (Supporting Information S1: Figure S1), consistency, and texture were noted, and a score assigned. The fecal score (1–5) was based on scoring systems for other zoo herbivores (Clauss et al. 2009; Taylor et al. 2013) (Supporting Information S1: Figure S2), with a score of 1–2 considered ideal. Two BR were transferred during the study period to different management settings and were sampled twice during the study, resulting in a total 84 BR samples. From some fecal samples that appeared particularly fresh, 1 g was weighed and mixed with 1mL of sulphuric acid (50% vol/vol) directly on site. A handful (50–300 g) of feces was sealed in a plastic bag, cooled below 6°C and frozen within 48 h. In one of the WR facilities, no quantitative information on diet items could be gathered, and no feed samples (only fecal samples) could be taken.

Dry feed samples were kept in paper bags in a cool (< 8°C), dry and dark place. Fecal samples were stored frozen below −18°C. After sample collection, feeds and feces were dried to constant weight in a drying oven at 60°C and then milled through a 1 mm screen.

Forages were analyzed for crude protein (CP), total ash (TA), neutral detergent fiber (NDF), acid detergent fiber (ADF) by NIRS (NIRS DS2500, FOSS, Denmark); for non‐forage feeds, these analytes were measured by wet chemistry (CP: AOAC no. 977.02; TA: 942.05; NDF‐ADF: 973.18) (AOAC 2023). Fecal samples were analysed with wet chemistry for total nitrogen, TA, ADF and acid‐detergent insoluble ash (ADIA; Hummel et al. 2011). For mineral analysis (Na, Ca, P, Fe, Cu, Zn), samples were microwave digested and analyzed by an inductively coupled plasma optical emission spectrometer (model Optima 8000, Perkin Elmer). The volatile fatty acids (VFA) were distinguished in seven fecal samples of WR and 13 of BR. Fecal samples preserved with sulphuric acid were topped up with distilled water to a volume of 5 mL and then centrifuged (10 min, 4000 g). In the collected supernatant VFA were determined in a gas chromatograph (Varian 450‐GC) equipped with a flame ionization detector (FID), using a DB‐FFAP column (30 m × 0.53 mm; film 1.00 μm; Agilent J&W GC Columns) and nitrogen as a carrier. The flow rate was set to 1 mL/min, and the temperatures for the column, injector, and detector were set to 90°C–205°C, 200°C, and 250°C, respectively.

Values for feedstuff not analysed in our study (mainly fresh feed) were taken from the literature (e.g., Schmidt et al. 2005).

### 2.1 Samples From Free‐Ranging Rhinos

Fecal samples < 24 h old from free‐ranging WR and BR in reserves in South Africa were collected between 2017 and 2018. Samples were dried by lyophilization or heating in an oven and were analysed with inductively coupled plasma‐optical emission spectrometry. Forty‐three samples of WR from eight reserves (only one of which, for *n* = 5 samples, provided artificial mineral licks) and ten samples of BR from three reserves (only one of which, for *n* = 3 samples, provided artificial mineral licks) were included in the evaluation. Within a reserve, repeated sampling of an individual could not be excluded but was considered unlikely as middens representing home ranges of different individuals were sampled. All reserves were located in South Africa. Note that these fecal samples are not directly linked to the available data on nutrient composition of diet items of free‐ranging rhinos.

### 2.2 Calculations

We differentiated between roughages (hays), compound feeds (generally, commercial pellets), single dry feeds (e.g., oat grains), vegetables, fruits and supplements (Table 1). Feed items which were not part of the daily diet, but given regularly under defined conditions (Supporting Information S1: Table S1), were not included in the calculations of diet composition. If several animals shared diet items, we calculated the proportional amount of the individual animals based on their metabolic body weights.

**Table 1:** Diet documentation, ration ingredients and practice of amount allocation in zoos keeping white rhinos (***Ceratotherium*** *simum*) and black rhinos (***Diceros*** *bicornis*).

		White rhino	Black rhino
		(23 facilities)	(22 facilities)
Feeding plan	Present	65% (15)	64% (14)
	Corresponded to diet fed on visit	80% (12)	93% (13)
Diet composition	Roughage based	100% (23)	100% (22)
	Grass hay	100% (23)	36% (8)
	Lucerne hay	4% (1)	96% (21)
	Browse	—	100% (22)
	Straw	9% (2)	5% (1)
	Hay cobs	9% (2)	13% (3)
	Haylage/silage	13% (3)	9% (2)
	Compound feeds	91% (21)	100% (22)
	Single feeds	30% (7)	36% (8)
	Oil	9% (2)	27% (6)
	Bread	4% (1)	18% (4)
	Linseed/linseed pellets	9% (2)	5% (1)
	Grains (oats/bran)	9% (2)	5% (1)
	Vegetables	4% (1)	59% (13)
	Carrots	4% (1)	55% (12)
	Brassicaceae	0% (0)	32% (7)
	Other green vegetables	0% (0)	46% (10)
	Potatoes/Sweet Potatoes	0% (0)	14% (3)
	Beet (seasonally)	4% (1)	9% (2)
	Fruit	4% (1)	50% (11)
	Apples	4% (1)	46% (10)
	Bananas	0% (0)	46% (10)
	Other fruits	0% (0)	18% (4)
Amount offered	Adjusting hay to previous day′s leftovers	70% (16)	82% (18)
	Ad libitum hay feeding	48% (11)	18% (4)
	Some leftovers desired	17% (4)	27% (6)
	Hay weighed	18% (4)	0% (0)
	Compound feeds weighed	9% (2)	32% (7)
	Scaled scoop/container for compound feeds	78% (18)	64% (14)
	Fixed amount of non‐forage feeds delivered daily by commissary	13% (3)	5% (1)

*Note:* Some institutions used both types of hay.

To estimate the diet composition, we followed an approach outlined by Flores‐Miyamoto et al. (2005). We assumed the following percentages of dry matter (DM) in fresh matter to calculate the total amount DM offered: dry roughages, compound feeds, single dry feeds 90%, vegetables and fruits 15%. We assumed that all compound feeds, single dry feeds, vegetables and fruit were consumed completely; on the days of the visits, basically no leftovers of these items were seen. The remaining difference to a total daily DM intake of 70 g/kg^0.75^ for WR and BR (Steuer et al. 2010) was assumed to be made up of the dry roughages. These estimates were also used to express the contribution of the individual diet items to the overall DM intake, and to estimate the nutrient composition of the overall diets. Additionally, these estimates were compared to the actual amount of forage feed on the day of the visit.

## 3 Results

### 3.1 Diet Plan and Diet Ingredients

**Figure 1:**
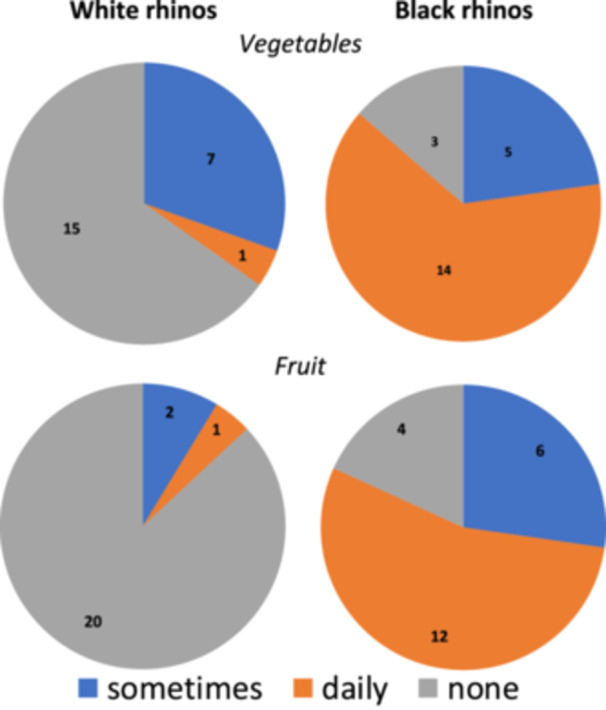
The frequency of feeding vegetables and fruit in 23 facilities with white rhinos (*Ceratotherium simun*) and 22 facilities with black rhinos (*Diceros bicornis*).

Only slightly more than half of all WR and BR facilities had a written feeding plan (Table 1), and the large majority of zoos (80% for WR, 93% for BR) adhered to it during the day of visit (Table 1). All institutions fed the rhinos a diet based on roughage (Table 1). In WR, a single institution offered nothing else and another one additionally hay cobs, whereas the majority of institutions fed their WR also a compound feed. In BR, all institutions used a compound feed and browse. The proportion of institutions that used vegetables and fruits was distinctively higher for BR than for WR (Figure 1).

#### 3.1.1 Dry Forages

**Figure 2:**
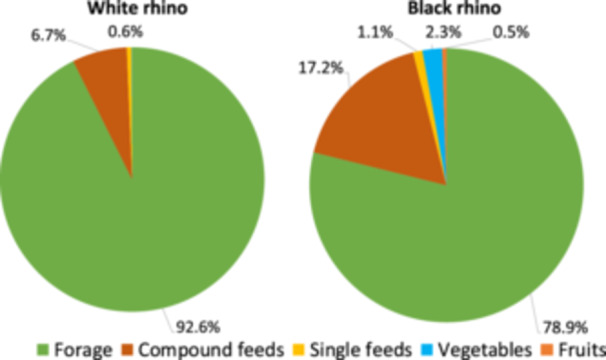
Average estimated diet composition (in % dry matter) in institutions with white rhinos (*Ceratotherium simum*) and black rhinos (*Diceros bicornis*). For an overview over the spread across individual institutions, see Supporting Information S1: Figure S3.

The proportion of forage DM in the total daily feed was significantly higher in WR than in BR (Figure 2, and Supporting Information S1: Figure S3). Only a single WR facility fed lucerne hay in addition to the commonly used grass hay; at this institution, the ratio grass: lucerne was about 10:1. Two institutions (9%) added straw to the hay, to reduce fecal water during the transition to summer pasture or keep the animals busy. Hay was not usually weighed at WR facilities.

Over half of BR institutions offered lucerne hay or lucerne haylage solely to their rhinos, whereas 32% (*n* = 7) fed roughage which consisted of a mix of lucerne and grass hay (Table 1). The ratios (lucerne: grass) were 3:2 (*n* = 1), 3:1 (*n* = 1), 2:1 (*n* = 3), 1:2 (*n* = 1) or varying daily (*n* = 1). One facility provided piles of straw and hay side‐by‐side.

#### 3.1.2 Fresh Forage

More than half (57%; *n* = 13) of the WR facilities offered fresh grass during the summer for various periods (Supporting Information S1: Table S2). Of the BR facilities, 23% (*n* = 5) offered fresh grass and another 23% (*n* = 5) offered fresh lucerne during summer (Supporting Information S1: Table S2). One of the latter gave a mix of lucerne and grass. Fresh leaves without wooden parts were fed in 18% (*n* = 4) when available. The latter was done in both BR facilities in the Netherlands, where rose leaves are easily available. In the other two zoos, the gardeners regularly brought in leaves that had been cut during hedgerow management and shrub pruning.

Of the 22 institutions holding BR, 91% (*n* = 20) provided at least some browse during the whole year depending on availability, including branches without foliage during winter. Willow was used most frequently for browse feeding (Supporting Information S1: Table S3 lists other common browse species). In 46% (*n* = 10) of the institutions, keepers stated to give various other browse species on an irregular basis. In 23% (*n* = 5) of the facilities, lists of inappropiate browse species existed. Poplar (*Populus* spp.), chestnut (*Castanea* spp.) and bramble (*Rubus* spp.) were among these because they were suspected to have contributed to health issues in single animals in the past. In another case oak (*Quercus* spp.) was fed only rarely in small amounts, because of a possible toxicity. BR were reported to not like certain browse species by 46% (*n* = 10) of the holders. Poplar (*Populus spp*.) was suggested as a disliked browse by 23% (*n* = 5) facilities.

BR institutions stated that they were able to feed branches usually every day in 41% (*n* = 9) of cases, a few times per week (1 to 3 days) in 14% (*n* = 3), and 4% (*n* = 1) obtained branches for the rhinos only occasionally. Another 41% (*n* = 9) of the BR holders could not specify the frequency of browse provision. The average amount of branches per day and animal was less than 5 branches in 41% (*n* = 9), 5–10 branches in 23% (*n* = 5) and more than 10 branches in 36% (*n* = 8) (Supporting Information S1: Table S3). Zoos that fed more browse also fed browse more frequently.

Most BR institutions offered browse on the floor or hung up (Supporting Information S1: Table S3). Some institutions (32%; *n* = 7) placed it leaning against the wall, or on items in the outside enclosure. Other institutions (14%; *n *= 3) stuck browse in the ground.

Several keepers reported that individual diseased or anorectic BR improved with abundant appropriate browse.

#### 3.1.3 Compound Feeds

The majority (91%; *n* = 21) of WR facilities used compound feeds in their diet. It was common practice to use pellets originally designed for other animal species (Supporting Information S1: Table S4). All of the BR holders used compound feeds. In 55% (*n* = 12), the compound feeds were especially designed for browsing species (Supporting Information S1: Table S4). Weighing the amount of compound feeds was more frequent in institutions with BR than with WR, but using some kind of measuring cup was generally common (Table 1).

#### 3.1.4 Vegetables and Fruits

For WR, only single institutions (4%; *n* = 1 each) fed apples or crushed carrots on a daily basis (Table 1). By contrast, half (50%; *n* = 11) of the BR facilities fed fruit every day. The most common fruit were apples and bananas, but melon, pear, orange and cranberry were also offered. Most (59%; *n* = 13) BR facilities gave their rhinos vegetables daily and 36% (*n* = 8) of them used varying kinds of vegetables each day depending on availability. Three institutions (14%) with BR fed boiled potatoes or sweet potatoes. All other vegetables were fed raw. Only 9% (*n* = 2) of the BR facilities fed the vegetables chopped into pieces. Fruits were usually fed whole.

#### 3.1.5 Single Feeds

In 30% (*n* = 7) WR facilities, single feeds comprised a part of the diet (Table 1). Linseed oil was suppelemented by 9% (*n* = 2), in one case only when considerer needed to improve skin condition, 9% (*n* = 2) fed linseed pellets and 4% (*n* = 1) whole linseed. Oats, wheat bran and bread were each fed by 4% (*n* = 1).

In 27% (*n* = 6) of BR facilities, oil was given regularly (Table 1). Oils used comprised ‘plant‐based omega‐3 oil′, olive oil, linseed oil, and grape oil. Other single feeds included bread (18%; *n* = 4), oats (5%; *n* = 1) and linseed pellets (5%; *n* = 1).

#### 3.1.6 Supplements

Single WR were supplemented with carotene bars or a supplementary feed for horses to enhance reproductive performance (Supporting Information S1: Table S5). The one facility which used zinc powder did this only temporarily in one single animal.

For the BR, vitamin E/selenium and biotin represented the most commonly used supplements (in 27% or *n* = 6 facilities, Supporting Information S1: Table S5). A vitamin E/selenium‐ preparation was given only twice a month for 3 days each time in one zoo; the other zoos supplemented it daily. Of the three (14%) institutions which supplemented vitamin C, all gave it three times a week, but one institution only seasonally during winter. Other usage of dietary supplements in BR are described in the Supplement.

#### 3.1.7 Salt Licks

Salt licks were offered by 35% (*n* = 8) of WR and 36% (*n* = 8) of BR holders (Supporting Information S1: Table S5). Most keepers indicated that both species of African rhinos are rarely if ever seen using them, except for two institutions in which the BR were observed using the salt licks repeatedly. The estimated Na content of the overall diets of these two institutions were 0.09%DM (i.e., a low Na content in our sample, cf. Table 2) and 0.24%DM (i.e., an average content).

**Table 2:** Analysed composition of different types of feeds used for white rhinos (*Ceratotherium simum*) and black rhinos (*Diceros bicornis*) as well as the calculated diet composition in visited zoologic facilities in Europe from the present study compared to values of their natural diet from the literature.

Type of feed	CP% DM	Total ash% DM	NDF% DM	ADF% DM	Na% DM	Ca% DM	P% DM	Fe mg/kg DM	Cu mg/kg DM	Zn mg/kg DM	Source
Grass hay^1^	7.8 ± 3.3)	7.0 (± 2.1)	60.5 (± 4.7)	34.3 (± 2.8)	0.1 (± 0.1)	0.9 (± 0.4)	0.2 (± 0.1)	163 (± 183)	5 (± 2)	27 (± 10)	
Lucerne hay^2^	13.1 (± 3.8)	8.6 (± 2)	45 (± 6.5)	38.8 (± 7.8)	0.1 (± 0.1)	1.3 (± 0.5)	0.3 (± 0.1)	176 (± 204)	9 (± 3)	30 (± 18)	
Haycobs^3^	11.8 (± 2.9)	9.6 (± 2.7)	13.5 (± 19.1)	7.5 (± 10.6)	0.0 (± 0.0)	1.4 (± 0.7)	0.2 (± 0.1)	1192 (± 772)	7 (± 2)	30 (± 5)	
Pellet (WR)^4^	15.5 (± 5.3)	10.2 (± 6.2)	33.7 (± 13.1)	19.4 (± 8.4)	0.7 (± 0.9)	1.9 (± 2.1)	0.7 (± 0.2)	464 (± 259)	44 (± 70)	200 (± 183)	
Pellet (BR)^5^	14.7 (± 1.6)	9.7 (± 1.7)	34.2 (± 14.4)	23.9 (± 12.1)	0.6 (± 0.3)	1.6 (± 0.6)	0.7 (± 0.2)	364 (± 141)	39 (± 27)	247 (± 210)	
Mineral feed^6^	18.3 (± 8.4)	29.4 (± 18.0)	5.8 (± 9.3)	3.5 (± 5.6)	2.2 (± 1.9)	0.5 (± 0.9)	1.5 (± 0.9)	1940 (± 1559)	312 (± 284)	1213 (± 1079)	
Single feed^7^	14.9 (± 10.5)	4.9 (± 2)	32.9 (± 18.3)	19.5 (± 16.2)	0.1 (± 0.1)	0.5 (± 0.9)	0.6 (± 0.5)	145 (± 145)	7(± 5)	48 (± 27)	
Other feed^8^	17.2 (± 5.6)	13.6 (± 10.8)	28.2 (± 22.5)	11.9 (± 12.4)	0.5 (± 0.6)	1.4 (± 1.6)	0.3 (± 0.1)	1597 (± 2253)	11 (± 9)	88 (± 69)	
Zoo diet (WR) (*n* = 114)	8 (± 2, 5‐14.)	7 (± 2, 4‐11)	57 (± 5, 34‐66)	33 (± 3, 22‐36)	0.2 (± 0.2, 0‐0.6)	0.5 (± 0.2, 0.3‐1.0)	0.3 (± 0.1, 0.1‐0.5)	189 (± 152, 38‐814)	7 (± 3, 4‐21)	41 (± 17, 18‐119)	
Zoo diet (BR) (*n* = 83)	12 (± 3, 3‐19)	8 (± 2, 5‐14)	45 (± 6, 31‐67)	35 (± 6, 20‐46)	0.2 (± 0.1, 0‐0.6)	1.1 (± 0.5, 0.3‐2.2)	0.3 (± 0.1, 0.1‐0.5)	174 (± 139, 45‐708)	13 (± 5, 2‐28)	56 (± 27, 12‐127)	
Natural diet	5 (± 1)	8 (± 1)	75 (± 1)	43 (± 1)	0.03 (± 0.01)	0.2 (± 0.1)	0.1 (± 0.0)	177 (± 49)	4 (± 1)	23 (± 8)	(1)
WR (grass)	6–13				0.1–0.5		0.1–0.2				(2)
Natural diet	4–11					0.7–3.2	0–0.2				(3)
BR (browse)	15	7	66	35	0.1						(4)
	8–11			36–48							(5)
	2–18	4‐41									(6)
	4–16	4‐13						12‐116	1–4	3–23	(7)
	12–23		37–57	25–41							(8)
	6–22		34–72	22–50	0–0.1	0.7–4.3	0.1–0.2	29–215	3–12	3–67	(9)
	17–22										(10)
	4–17		31–72								(11)
	5–11		37–61	25–46	0–0.3	0.5–1.8	0–0.1	41–216	3–6	12–50	(12)

*Note:* n: ^1^29, ^2^23, ^3^6, ^4^25, ^5^23, ^6^5, ^7^11, ^8^other included trainings pellets, two medicinal feed additives and two grain‐based feed mixtures.

#### 3.1.8 Extras

Most institutions used extra food for special occasions, which was not listed in the feeding plan (Supporting Information S1: Table S1). Amounts and frequencies varied widely within and between institutions. The degree to which these feed items contribute to the daily feed quantity could not be determined, as most of them were not measured or documented regarding their amount. Dietary care for special cases are listed in the Supplement.

#### 3.1.9 Feeding Practice

Most facilities holding WR offered two feedings per day (Supporting Information S1: Table S6). One institution provided a third portion of roughage at noon. In another one, roughage feeding was controlled by automatic feeders, so the animals received hay at least eight times a day at various feeders in the enclosures during day‐ and nighttime. Additionally, the keepers of this zoo sometimes placed roughage manually in some inside enclosures. All WR holders offered feed in the inside as well as in the outside enclosures (Supporting Information S1: Table S6). All of them offered hay on the floor or the ground. However, 26% (*n* = 6) fed hay only from certain concrete or stone spots to avoid sand or soil intake. Only 13% (*n* = 3) of WR holders used hayracks. Group feeding occurred in all institutions with WR (Supporting Information S1: Table S6).

In BR facilities, it was also common feed twice per day (Supporting Information S1: Table S6). In 9% (*n* = 2) of institutions, there was a third feeding at noon, and in one zoo (5%), forage was refilled several times a day following no fixed schedule or regularity. In one zoo (5%) the feeding frequency was reduced to one single feeding in the morning during summer. In some facilities, browse was not necessarily put in the outside enclosure at the same time as the other food and hence was available at different times each day. All BR holders fed the rhinos in the inside enclosures, and in 77% (*n* = 17) it was common to also feed roughage in the outside enclosures. The other 23% (*n* = 5) occasionally gave browse or vegetables in the outside enclosures. Whereas 91% (*n* = 20) of facilities fed hay on the ground, only 14% (*n* = 3) had a dedicated stone or concrete spot for this. Three holders (14%) placed lucerne hay into hung‐up items like barrels or wheels, and one (5%) in a hay net, which was only frequented by one of the two rhinos at that facility. Feeding in a group or pair occurred only in 27% (*n* = 6) of institutions holding BR and was limited exclusively to the outside enclosure (Supporting Information S1: Table S6).

Rhinos were included in visitor feeding progams by 13% (*n* = 3) of WR and 32% (*n* = 7) of BR holders, including commented feedings in front of visitor groups, or special experiences for single visitors who were allowed to feed the rhinos by hand (mostly vegetables and browse, sometimes fruits). For the interactions with single visitors, all zoos involved stated to use only individual rhinos selected based on their character and daily mood.

During visits, it was observed several times that dominant female WR chased bulls away from feeding sites when they approached very close. Multiple females of both species, on the other hand, fed at the same feeding spot peacefully side by side.

### 3.2 Quantities and Proportions

The average ±SD daily amount of non‐forage DM in facilities with WR was 1.39 ± 1.57 kg DM, and the average assumed proportion of forage 93 ± 8% (Supporting Information S1: Table S7, Figure 2). On the day of the visit, 11% (*n* = 13) WR received less forage than their assumed consumption, in 16% (*n* = 19) the weighed amount was less than the assumption but there was additional forage in the outside enclosure, in 14% (*n* = 16) the actual amount of forage corresponded to the assumption with a margin of ±3 kg. For 44% (*n* = 51) of the animals, the amount offered exceeded the assumed consumption distinctively, and in 15% (*n* = 17) the amount of hay offered could not be quantified reliably. When comparing the sexes, the mean daily amount of compound feeds was 5.3 ± 5.5 g/kg^0.75^ in female and 6.0 ± 5.1 g/kg^0.75^ in male WR, or 0.4 vs. 0.5% of body mass.

In institutions with BR, the average daily amount of non‐forage was higher at 3.06 ± 3.54 kg DM, and the average assumed proportion of forage lower at 79 ± 24% compared to WR (Supporting Information S1: Table S7, Figure 2). Only 1% (*n* = 1) of BR received less forage than the assumed consumption on the day of the visit, 22% (*n* = 18) received about the assumed amount with a margin of ±3 kg, and 77% (*n* = 64) received more. Female BR received a daily allotment of compound feeds at 9.4 ± 7.5 g/kg^0.75^ and males at 11.9 ± 8.7 g/kg^0.75^, or 0.8 vs. 1.3% of body mass.

### 3.3 Feed and Diet Nutrient Composition

As expected, grass hay contained less protein, less calcium and more NDF than lucerne hay (Table 2). On average, iron levels in both kinds of hay were below 200 mg/kg, but there was a wide range for the lucerne hays (30–869 mg/kg DM). Compound feeds used in both species had about 30% NDF and 20% ADF, iron levels higher than 350 mg/kg, and higher levels of other minerals than the forages (Table 2).

The diets fed to WR were higher in NDF and iron compared to those fed to BR, but both species received diets with average iron levels below 200 mg/kg (Table 2). Compared to data on individual diet items from free‐ranging animals, WR zoo diets were lower in NDF and ADF, and lower in all measured minerals except iron (Table 2). Zoo BR diets had levels of protein, ash, NDF and ADF in the range of natural diet items; zoo diets were also within these ranges for calcium, iron and zinc, and higher for sodium and phosphorus. Average iron levels in zoo BR diets were at the higher end of the range for natural diet items, and the iron range in the zoo diets exceeded that of the natural diet items by far (Table 2). The highest dietary iron concentration in WR of the present study was 814 mg/kg DM and occurred in one of the two old rhinos described under exceptional cases in the Supplement, which received a higher amount of compound feeds due to geriatric tooth problems. The second highest dietary iron levels in WR was 502 mg/kg DM, and diets of 26 animals in total exceeded the threshold recommended for BR of 300 mg/kg DM (Sullivan et al. 2020). Most of the BR had dietary iron concentrations below the recommended 300 mg/kg DM, and only diets of nine animals from four different zoos exceeded this value. Additionally, there were 26 BR from eight zoos which had estimated dietary iron levels below 100 mg/kg DM. The lowest dietary iron levels occurred in the BR listed under exceptional cases, which did not receive any kind of compound feeds and only leafy green vegetables (43.8 mg/kg DM).

### 3.4 Fecal Scores and Measurements

A total of 85 WR and 80 BR fecal samples were evaluated. The feces of some of the animals were partly or totally trampled, so that in some cases it was not possible to assign a score. For WR, 67% (*n* = 57) were individual samples; the others were group samples. The majority of sampled WR facilities was given a fecal score of two (91%; *n* = 21) and a fecal colour of medium brown (91%; *n* = 21) (Supporting Information S1: Table S8). For BR, 95% (*n* = 76) were individual samples. Most of the BR facilities were assigned a score of three (59%; *n* = 13) and the colour medium brown (73%; *n* = 16; Supporting Information S1: Table S8).

On average, BR feces had higher total fecal nitrogen, ADF and copper than WR feces, but lower feces averages for silicates (ADIA), zinc and iron (Table 3). Compared to feces in natural habitats, the zoo feces of both species were lower in iron; zoo BR had higher levels of fecal nitrogen, copper and zinc (Table 3). In particular, feces of zoo BR had much higher silicate levels than free‐ranging conspecifics, and were actually of a magnitude observed in free‐ranging WR (Table 3).

**Table 3:** Analysed composition of feces of white rhinos (*Ceratotherium simum*) and black rhinos (*Diceros bicornis*) in visited zoologic facilities in Europe from the present study compared to values of animals from natural habitats. Values in mean (± SD; min‐max).

Type of feed	N% DM	Total ash% DM	ADF% DM	ADIA% DM	Na% DM	Ca% DM	P% DM	Fe mg/kg DM	Cu mg/kg DM	Zn mg/kg DM	Source
White rhino Zoo (*n* = 117)	1.4 (± 0.2, 1.1–1.9)	17 (± 7, 9–32)	40 (± 5, 31–48)	11 (± 7, 4–28)	0.2 (± 0.1, 0–0.6)	0.4 (± 0.2, 0.2–0.8)	0.5 (± 0.2, 0.3–0.8)	1618 (± 1099, 378–4918)	18 (± 7, 10–33)	98 (± 49, 49–246)	
Black rhinoZoo (*n* = 84)	1.8 (± 0.5, 1.0–2.8)	13 (± 6, 6–31)	51 (± 6, 39–62)	7 (± 6, 1–22)	0.3 (± 0.2, 0–0.6)	0.5 (± 0.2, 0.2–0.9)	0.6 (± 0.2, 0.3–1.1)	1014 (± 771, 234–3265)	25 (± 10, 10–45)	127 (± 50, 72–258)	
Whinte rhino nat.	1.3		32								(5)
	1.6 (± 0.2, 1.4–2.0)				0.2 (± 0.1, 0–0.5)	0.5 (0.2, 0.1–1.2)	0.3 (± 0.1, 0.1–0.5)	3550 (± 3126, 142–15030)	14 (± 6, 5–33)	56 (± 17, 19–98)	(6)
				7.5 ± 1.3							(7)
Black rhino nat.											
	1.0–1.5	7–14	50–58								(1)
	0.9	6				2.1–6.5					(2)
						1.0 ± 0.4	1.3 ± 0.8		12 ± 6	52 ± 38	(3)
	1.3		51								(5)
				1.7 ± 0.6							(7)
					0.2 (± 0.1, 0–0.3)	1.5 (± 0.3, 0.9–2.0)	0.2 (± 0, 0.2–0.3)	3775 (± 5055, 400–15660)	20 (± 12, 6–42)	35 (± 9, 26–56)	(6)

*Note:* (1) (Hall‐Martin et al. 1982), (2) (Loutit et al. 1987), (3) (Okita‐Ouma et al. 2021), (5) (Codron et al. 2007), (6) Abraham (pers. comm.), (7) (Hummel et al. 2011).

A preservation error affected all fecal samples stored for volatile fatty acids; we did not consider the resulting absolute values reliable. However, the ratio of acetate:propionate was higher in the WR at 189 ( ± 63) than in the BR at 44 ( ± 23).

## 4 Discussion

The present study provides an overview of current feeding practices for African rhinos kept at European zoos and identifies differences in the dietary management between WR and BR. Although both rhino species are fed like herbivores with the majority of the diet originating from forage, BR receive a higher proportion of pelleted feed, produce and additional items. This is also reflected in differences in fecal measurements between the species. To our surprise, the data dit not indicate the systematic overprovision of European zoo BR with the excessive absolute iron loads we had expected, raising the question about other causes for the ubiquity of iron overload in the species (Radeke‐Auer et al. 2023), and shifting the dietary focus towards both subtly increased absolute iron levels and questions of iron bioavailability. The findings also point towards a dilemma in choosing the adequate forage for BR. Before these points are discussed in more detail, some limitations of the present study need to be mentioned.

### 4.1 Limitations of the Study

Quantifying the diet of animals that received certain diet items for ad libitum consumption, such as hay or browse, is inherently challenging. The gold standard would be to weigh the amount of forage offered and the leftovers on the next day, and repeat this procedure consistently for about a week to obtain a reliable picture of the intake of groups or individuals. While this is commonly done in digestion studies that focus on a small number of animals in a few institutions and that require a direct match of diet nutrient intake and fecal nutrient excretion (Clauss et al. 2006; Steuer et al. 2010), this is too labour intensive for both, a larger‐scale survey or a request to put to the participating zoos. Also, even when determining individual intake with precision for a week, extrapolating this to a lifetime diet, as is considered relevant for long‐term issues like IOD, will be similarly speculative as when the intake is only plausibly estimated. A simple solution to nevertheless roughly estimate the overall diet composition, as performed here based on Flores‐Miyamoto et al. (2005), is to assume a certain dry matter intake across animals—here based on estimates of metabolic body weight—, assume a complete intake of the non‐foreage items that are typically not offered for ad libitum consumption, and then calculate the amount of ingested forage as the difference between the two. Such an approach inherently ignores individual differences between animals and should not be used to compare specific specimens, but may yield a pragmatic overview over a larger population. The fact that in the majority of cases, there was no relevant discrepancy between the estimated amount of forage ingested and the amount actually offered—which was often in excess of the estimate, suggestive of provision for ad libitum consumption—gives some indication that this approach might be considered representative.

A particular challenge is the quantification of browse offered to the animals. In the absence of logistical means for a more thorough approach, the provision of browse was only estimated by information on the frequency and the number of branches.

With respect to nutrient composition of diet items, a more comprehensive assessment might be desirable, including, for example, measures of starch and water soluble carbohydrates or silica content, to be less reliant on literature assumptions. As for spot fecal samples, they are inherently less valuable than data derived from total fecal collection over a longer period, ideally in parallel to a detailed intake measurement.

Any comparison with data from natural habitats is constrained by limited sample sizes and localities; that in most cases, individual diet item composition is known rather than the composition of the average diet; and that to date, no data exists that links the natural diet to the feces of the same individuals. Rather, so far, individual fecal samples are used as proxies for the assessment of habitat nutrient availability (e.g., Okita‐Ouma et al. 2021). A large‐scale study on nutrient composition of natural diets and feces across the whole habitat range would evidently be welcome.

### 4.2 Diet Ingredients and Proportions

For zoo herbivores, the generic recommendation is that the majority of the diet should be composed of dried or fresh forages such as hay, supplemented with a (commercial) compound feed that ensures mineral and vitamin provision, to balance the diet based on general knowledge of the typical composition of forages (in general: Lintzenich and Ward 1997; for rhinos: Dierenfeld 1999; Clauss and Hatt 2006; Clauss et al. 2012; Sullivan and Valdes 2019). With 93% and 79% of the dietary dry matter consisting of forages (Figure 2), rhino diets in general follow this recommendation. However, the difference between the two species appears striking: compared to WR, BR receive higher proportions of pelleted compound feeds, fruit and vegetables, and other non‐forage items (Figure 2), which are all easier to digest than forage. This corresponds to the higher levels of propionate in BR as compared to other rhino species in North American zoos reported recently (Roth et al. 2019; Cersosimo et al. 2022) that were also qualitatively confirmed in the present study. Additionally, higher concentrations of total fecal nitrogen—an indicator for diet digestibility (Steuer et al. 2014)—also pointed towards diets of higher digestibility in BR (Table 3). Possibly, the somewhat softer and darker feces in BR (Supporting Information S1: Table S8) are also related to this difference.

This observation leads to two questions: what is the reason for this difference, and does this difference have relevance for the putative health of BR? The current European Best Practie Guidelines for BR do not recommend the use of fruit and vegetables, and recommend a pelleted compound feed at a maximum of 10% of the diet (Pilgrim and Biddle 2020). Both recommendations are evidently not followed by many BR facilities (Figure 2). On the one hand, this could be a cause for a general tendency towards obesity in the European BR population. Obesity has been suggested as a predisposing factor for disease syndromes typical for BR, including iron overload disorder (Schook et al. 2015). On the other hand, zoo managers might include more pelleted feed and produce in the diets of BR as a reaction to a general propensity of many BR to present a lower body condition (Clauss and Hatt 2006). To answer this question, information on the body condition of the current European zoo rhino population is needed.

Whether the increased use of pelleted feed and fruits and vegetables in BR is due to a species‐specific tradition, possibly also caused by a lack of suitable forage alternatives for browse, cannot be answered here. In a similar vein, the observation that males of both rhino species were on average allocated more compound feed than the respective females (both on a body weight and a metabolic body weight basis) is another indication of systematic yet unknown motivations for feeding practices.

### 4.3 Diet Nutrient Composition

With their high reliance on forages, zoo diets mimicked the macronutrient composition of free‐ranging WR and BR diets reasonably well, especially with respect to fiber levels (Table 2). Similarly, fecal fiber levels did not indicate a general shortage of dietary fiber compared to free‐ranging specimens (Table 3). Thus, in terms of diet ingredients and in the absence of actual intake data, the diet composition documented in the present study does not per se suggest a high likelihood for widespread obesity in the European BR population. Again, an overview over the current populations' body condition would be welcome. Generally, the diets of both species were higher in Na, P, Cu and Zn than concentrations reported in the wild (Table 2), suggesting that the concept of a mineral safety provided by compound feeds is valid. This is also reflected in higher fecal concentrations of these minerals in the zoo populations compared to free‐ranging specimens (Table 3). This difference matches the observation that free‐ranging rhinos make ample use of salt licks put out in nature reserves (Abraham et al. 2023), whereas according to the keepers interviewed during the present study, European zoo rhinos—with their higher dietary Na concentrations—hardly used the salt licks provided. It might be worthwhile, in the future, to consider a reduction of Na in zoo compound feeds to make salt licks more attractive to zoo herbivores, and thus provide zoo managers with an additional tool to make enclosures spatially and temporally more interesting by providing salt licks at different locations and frequencies. Of course, such an approach would have to be weighed carefully against institution‐specific challenges in maintaining a planned salt lick regime.

For Ca, which is typically provided mainly via the forages, higher levels in zoo as compared to free‐ranging WR was evident (Table 2). For BR, higher Ca levels have been observed in natural diets—a typical feature of dicotyledonous (browse) plant food (Table 2). There is no indication that the somewhat lower levels of Ca in zoos are problematic. Hindgut fermenters such as rhinos absorb the a large proportion of Ca from the gut and excrete it via kidneys (Clauss et al. 2005b; Clauss, Castell, et al. 2007; Hagen et al. 2015; Liesegang et al. 2024). Lower (but adequate) levels of dietary Ca might simply represent a reduced channelling of Ca into urine.

For phosphorus, the average dietary provision was also higher than that reported in the wild for both species (Table 2). Hypophosphatemia has been of concern in BR, also in connection with iron overload disorder (Sullivan et al. 2020). The present data does not suggest a general dietary mismanagement as a potential cause, even when considering that a part of the phosphorus in those compound feeds that are based on cereals may be phytate and hence of limited availability (e.g., Humer and Zebeli 2015).

### 4.4 Dietary Iron

BR are susceptible to iron overload disorder (IOD) whereas WR are not (reviewed in Clauss and Paglia 2012). A thorough review on all aspects of IOD is given by Sullivan et al. (2020). There are clear recommendations to limit the dietary iron intake in BR, with a target range of 50–100 mg/kg dry matter (Clauss et al. 2012) that may be difficult to attain, and a more practically feasible one of 300 mg/kg dry matter (Sullivan et al. 2020).

Matching these recommendations, the estimated dietary iron levels were lower in BR than in WR, and at an average of 174 mg/kg dry matter, cannot be considered excessive. This average is within the range of diet items of free‐ranging specimens (Table 2). Similarly, Ricketts et al. (2021) showed that a BR diet at a UK facility was below the recommended threshold and resembled that of free‐range diets. Additionally, a comparison of fecal iron levels from the animals of the present study and free‐ranging specimens does not suggest unnaturally high iron contents in zoo diets, either (Table 3). Should IOD be the direct effect of excessive dietary iron intakes, one would only expect BR from specific facilities that feed high‐iron diets to be affected. By contrast, IOD is ubiquitous in the European (and possibly the global) BR zoo population (Radeke‐Auer et al. 2023).

**Figure 3:**
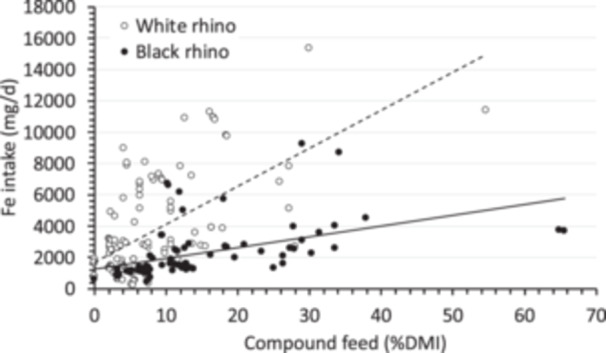
Iron intake of white rhinos (*Ceratotherium simum*) and black rhinos (*Diceros bicornis*) in correlation to the proportion of compound feed of the total dry matter intake (DMI).

Nevertheless, irrespective of the underlying etiopathology, iron that manifests as IOD must derive from dietary iron, and therefore, limiting dietary iron is one of the rational measures in the attempt to limit IOD (Clauss and Paglia 2012; Sullivan et al. 2020). The use and composition of pelleted compound feeds must continuously be evaluated critically– and a reduction to the level provided to WR might be more appropriate (in combination with adequate forage). In the present data set, a higher proportion of the (pelleted) compound feed in the overall diet was related to a higher estimated iron intake (Figure 3). Notably, during the survey, there was no indication that diets were composed deliberately to maintain low iron levels—they just turned out that way, and should give no cause for complacency.

Other dietary factors—in particular, the difference in iron‐binding tannins that occur in browse but not in common zoo diet items—may play a role in the susceptibility of BR to iron overload. These substances most likely reduce the bioavailability of iron in browse (Lavin 2012; Sullivan et al. 2020), which also means that a comparison of sheer iron levels between natural browse and zoo diets is not wholly adequate. Thus, the potential for differences of iron bioavailability between feeds to affect dietary iron uptake in zoo rhinos should not be overlooked.

However, the discrepancy between the ubiquity of the disorder and the moderate levels of dietary iron detected in the present study suggests that non‐dietary causes of IOD should also be considered. Amongst possible candidates, the general stress susceptibility of BR (as compared to WR or greater one‐horned rhino) has been mentioned (Radeke‐Auer et al. 2023). Nevertheless, the endeavour to keep dietary iron levels low in BR must continue; this should include an assessment of supplements that might contain high levels of iron or—like vitamin C—can distinctively increase its availability (Sullivan et al. 2020).

### 4.5 Choice of Forage

Herbivores should generally be fed diets consisting mainly of forages. The two most commonly available conserved forage types used in European zoos are varieties of grass and lucerne hay. Grass hay should be the forage of choice for animals whose natural diet consists of grass, such as WR, and this is what we found in the European WR population. For species naturally adapted to browse, grass hay generally has two problematic properties: (i) several browsing species, such as browsing ruminants or tapirs (*Tapirus* spp.) do not accept grass hay in sufficient amounts (Foose 1982; Clauss et al. 2003a), possibly due to the fact that their teeth cannot chew grass efficiently (Hummel et al. 2008), making an alternative forage necessary—typically, lucerne hay. (ii) grasses contain silica bodies called phytoliths that wear down teeth (Hummel et al. 2011; Müller et al. 2014; Martin et al. 2019). The observation that both browsing ruminants (Clauss, Franz‐Odendaal, et al. 2007; Kaiser et al. 2009) and browsing rhinos (Taylor et al. 2014) have higher tooth wear in zoos compared to free‐ranging specimens has been suggested to be caused by the use of phytolith‐containing grass products in zoo diets—grass hay or fresh grass, but also grass meal or cereal byproducts such as hulls in compound feeds. In contrast to tapirs or browsing ruminants, BR generally do accept grass hay well (Foose 1982; Grant et al. 2002; Clauss and Hatt 2006; Steuer et al. 2010; Cersosimo et al. 2022, and the many observations during the present study), but this may therefore adversely affect their teeth. In the present study, the feces of zoo‐managed BR had silica levels that were much higher than those of free‐ranging specimens; actually, their fecal silica levels were of a magnitude reported for free‐ranging WR (Table 3). Such high levels of abrasives could well explain the recent finding in the European BR population of excessive tooth wear in adult BR (Radeke‐Auer et al. 2023). Based on these considerations, the use of grass hay and grass products should be reconsidered in BR, and special care should be taken to avoid contamination of feeds with abrasive substances by offering forages directly on soil.

However, more aspects have to be considered when selecting an appropriate forage. As a dicotyledonous plant, lucerne hardly contains silicaceous phytoliths and therefore should not contribute to tooth wear to the same extent as grass hay (Clauss, Franz‐Odendaal, et al. 2007). This may occasionally differ if lucerne is harvested very close to the ground so that grit and stones are included in the baled product (M. Clauss, pers. obs.). Forage quality control, and ideally contact with harvesters, is recommended. Lucerne is also high in protein and calcium and thus resembles the nutrient composition of browse on a dry matter basis (Table 2). The concern that the protein and calcium content of lucerne may represent a problem for BR (Sullivan and Valdes 2019) that are devoted browsers therefore seems difficult to understand, at least for European varieties of lucerne investigated here. It should be noted that commonly available lucerne hay varieties may differ distinctively between the continents (Clauss and Dierenfeld 2008). Browsers that do not accept grass hay typically accept lucerne hay more readily (Clauss and Dierenfeld 2008). However, lucerne hay has been reported to often be high in iron – something to avoid in BR; therefore, a 1:1 mix of lucerne and grass hay has been recommended traditionally (Clauss et al. 2012; Sullivan and Valdes 2019). By contrast, the lucerne hays surveyed in the present study (Table 2) as well as that of another recent study in the UK (Ricketts et al. 2021) do not indicate consistently high iron levels of lucerne hay. However, to be sure, a zoo would have to analyze a batch of lucerne hay for its iron content. Another issue with lucerne hay is the content of phytoestrogens that have been implicated in reduced fertility in WR (Tubbs et al. 2016). To what extent this affects BR is unknown.

Given these dilemmata when choosing between grass and lucerne hay, it appears evident that a major focus in feeding BR should be on the forage they tolerate best—browse. Browse is typically difficult to quantify, which should not lead to a lack of attention to the amounts actually fed. Recently, Ricketts et al. (2021) documented that browse represented up to 27% of total dry matter intake in BR at a UK institution. Using equations that link the total mass of a branch and the mass of its leaves to its diameter at cutting (Clauss et al. 2003b), a 1000 kg BR with an assumed daily dry matter intake of about 12.4 kg would require 15 branches with a diameter of 4 cm to achieve 50% of its daily dry matter intake from browse, which would translate into a total fresh branch mass of 70 kg (with an estimated fresh leaf mass of 17 kg) per day. At a diameter of only 3 cm, this would require 27 branches of a total mass of 60 kg (and again 17 kg fresh leaf mass). Even without knowing the diameter of the branches used in the present study, it is clear that the majority of BR facilities did not achieve such values at less than 10 branches per day. Additionally, it appears doubtful that those feeding more than 10 branches per BR come close to these values. Clearly, the resulting recommendation must be the same as stated repeatedly before (Clauss et al. 2012; Sullivan et al. 2020) that browse provision be taken very seriously by BR holders, and that efforts must be increased to guarantee a daily provision of a large amount—for example, 15 branches at a diameter at the point of cutting of 4 cm. Including high amounts of browse should not lead to a restriction of ad libitum forage components of the diet, to allow the animals to adjust their intake level to the digestible energy content, and may require adjustments in supplements used.

Alternative dicotyledonous hays have been named in the literature, in particular sainfoin (*Onobrychis viciifolia*) and lespedeza (*Lespedeza cuneata*) (Clauss et al. 2012). However, none of the facilities visited in this study used hay of these plants, possibly due to their very restricted commercial availability. For progress in BR husbandry, not only increased provision of browse, but also the routine cultivation of such plants for zoos would be important.

### 4.6 Feeding Practice

Several other observations were made in the present study that deserve to be mentioned as they are in conflict with current feeding recommendations. Vitamin C should not be supplemented to BR, due to its iron‐absorption enhancing effect (Sullivan et al. 2020). In particular, the feeding of domestic fruit, tuber vegetables, cereals or bread to large herbivores is not recommended (Clauss and Dierenfeld 2008). Food should never be offered on a substrate that could be ingested accidentally and cause health issues, such as dental wear (Martin Jurado et al. 2008). Involuntary ingestion of soil in a zoo or a free‐range setting may also contribute to nominal iron intake (though, possibly, not in a particularly bioavailable form) and may therefore influence fecal iron levels. Whether the use of oil is beneficial should be critically assessed, as well as the use of other individual diet supplements. In theory, a forage‐based diet combined with a forage‐based compound feed (i.e., no use of cereals, cereal‐based compound feeds, or bread) should not require supplementation for omega‐three fatty acids (Clauss, Grum, et al. 2007; Clauss et al. 2008). By default, food should be offered to group‐kept animals at multiple locations so that agonistic encounters are avoided (Schmidt and Sachser 1996). Putative beneficial effects of using methods to prolong feed intake, such as multiple feeding locations, possibly combined with automated hay delivery, should be further explored.

## 5 Conclusions

Due to the absence of correlated health parameters, we cannot derive evidence‐driven feeding recommendations from this study. We conclude that WR, in general, are fed in accordance to recommendations in the surveyed population, with an appropriate forage (grass or grass hay) representing the large majority of the diet, supplemented with a compound feed. By contrast, compound feeds are used in higher proportions in BR, and BR also receive more often diet items not recommended for this or other herbivore species such as fruit, bread, or potatoes. For BR, the choice of an appropriate forage is particularly difficult, due to perceived disadvantages of grass hay (in terms of dental health due to abrasives, elevated in the feces of zoo BR compared to free‐ranging specimens), and putative disadvantages of lucerne hay (e.g., in terms of iron contents). Therefore, we recommend that BR holders put an modification of their dietary management on the short‐term agenda, including institutionalized effort to ensure a high daily amount of browse for BR as a husbandry default, not a facultative add‐on. Additionally, BR facilities should ensure access to high‐quality, low‐iron lucerne hay, fresh lucerne, or lucerne haylage, and combine efforts to obtain alternative forages like sainfoin hay for their animals.

## Ethics Statement

The authors have nothing to report.

## Conflicts of Interest

The authors declare no conflicts of interest.

## Supporting information


**Figure S1:** Fecal colours in white rhinos (*C. simum*) and black rhinos (*D. bicornis*). **Figure S2:** Scoring system for fecal consistency for white rhinos (*C. simum*) and black rhinos (*D. bicornis*). **Figure S3:** Estimated diet composition (in % dry matter) in individual institutions with white rhinos (*C. simum*) and black rhinos (*D. bicornis*). **Table S1:** Feed items and amounts used as additional extras for the rhinos on the listed occasions in facilities with white rhinos (*C. simum*) and black rhinos (*D. bicornis*). **Table S2:** The use of fresh grass and fresh lucerne seasonally during summer in zoos keeping white rhinos (*C. simum*) and black rhinos (*D. bicornis*). **Table S3:** Browse species used as feed items, browse sources and winter browse options in visited zoos keeping black rhinos (*D. bicornis*). **Table S4:** Compound feeds in zoos keeping white rhinos (*C. simum*) and black rhinos (*D. bicornis*)*. **Table S5:** Salt and other supplements fed in zoos keeping white rhinos (*C. simum*) and black rhinos (*D. bicornis*). **Table S6:** Frequency of feeding and feeding practices and locations in facilities with white rhinos (*C. simum*) and black rhinos (*D. bicornis*). **Table S7:** Nutritional components on average in the diet of visited institutions with white rhinos (*C. simum*) and black rhinos (*D. bicornis*). **Table S8:** Average values of fecal score and colour in facilities with white rhinos (*C. simum*) and black rhinos (*D. bicornis*).

## Data Availability

The data that support the findings of this study are available from the corresponding author upon reasonable request.
