# Supplementary material for: Feeding Management of African Rhinos (Ceratotherium simum, Diceros bicornis) in European Zoos
Source: Zoo Biol. 2025 Oct 11;45(2):109–23. doi: 10.1002/zoo.70031 (PMC13051751; doi:10.1002/zoo.70031)
Supplement: Supplementary file 1 — Figure S1: Fecal colours in white rhinos (C. simum) and black rhinos (D. bicornis). Figure S2: Scoring system for fecal consistency for white rhinos (C. simum) and black rhinos (D. bicornis). Figure S3: Estimated diet composition (in % dry matter) in individual institutions with white rhinos (C. simum) and black rhinos (D. bicornis). Table S1: Feed items and amounts used as additional extras for the rhinos on the listed occasions in facilities with white rhinos (C. simum) and black rhinos (D. bicornis). Table S2: The use of fresh grass and fresh lucerne seasonally during summer in zoos keeping white rhinos (C. simum) and black rhinos (D. bicornis). Table S3: Browse species used as feed items, browse sources and winter browse options in visited zoos keeping black rhinos (D. bicornis). Table S4: Compound feeds in zoos keeping white rhinos (C. simum) and black rhinos (D. bicornis)*. Table S5: Salt and other supplements fed in zoos keeping white rhinos (C. simum) and black rhinos (D. bicornis). Table S6: Frequency of feeding and feeding practices and locations in facilities with white rhinos (C. simum) and black rhinos (D. bicornis). Table S7: Nutritional components on average in the diet of visited institutions with white rhinos (C. simum) and black rhinos (D. bicornis). Table S8: Average values of fecal score and colour in facilities with white rhinos (C. simum) and black rhinos (D. bicornis). [file ZOO-45-109-s001.docx]

*Supplementary material*

**Feeding management of African rhinos (*Ceratotherium simum*, *Diceros bicornis*) in European zoos**

Gila Sauspeter, Marcus Clauss, Sylvia Ortmann, Andrew J. Abraham, Rebecca Biddle, Lars Versteege, Marcin Przybyło

**Supplementary methods**


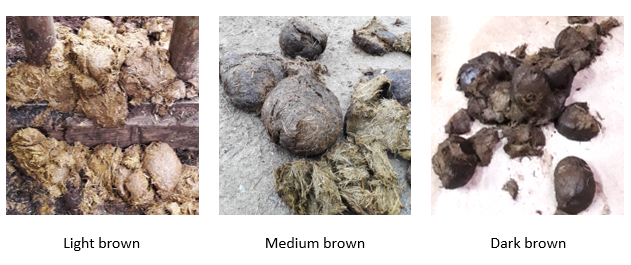


**Figure S1.** Fecal colours in white rhinos (*C. simum*) and black rhinos (*D. bicornis*).

**
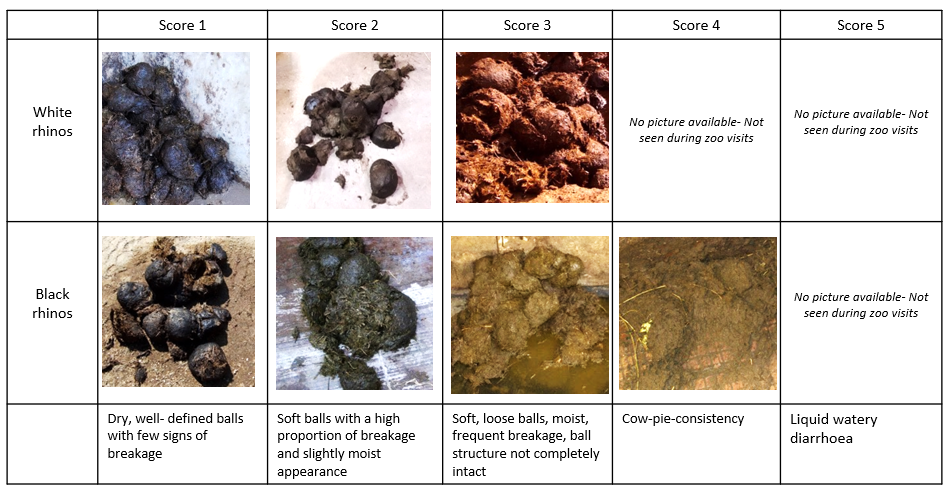
**

**Figure S2.** Scoring system for fecal consistency for white rhinos (*C. simum*) and black rhinos (*D. bicornis*).

**Supplementary results**

**Figure S3.** Estimated diet composition (in % dry matter) in individual institutions with white rhinos (*C. simum*) and black rhinos (*D. bicornis*). For an overview over the averages per species, see Fig. 2. Note that in one white rhino facility, no quantitative diet information was available.

***Supplement use in black rhinos***

One facility supplemented a multivitamin supplement only seasonally during winter. Another institution fed four different supplements for horses, one with psyllium husks against sand colic, one for liver and kidney protection, one for gastric mucous membrane protection and one for general performance enhancement. The rhinos had received these supplements for some time and were showing good health, which was why the administration was currently maintained. Single rhinos received nutritional supplements for horses due to their health conditions, including one time a liver protective and one time a joint protective supplementary feed.

***Exceptional cases***

There were two white and four black rhinos which were each fed individually adjusted diets due to health conditions at the time of the visit. These adjustments were excluded from the analyses above. Two female white rhinos in different facilities, both over 50 years old, had problems with ingesting high quantities of their usual roughage, presumably due to tooth problems, so both were offered less of the usual hay and chopped hay instead. Furthermore, one was offered more pellets and extra hay cobs, and the other one a mix of mash, hay cobs and supplements for horses.

There were two black rhinos in different institutions with presumed tooth problems, too, which led to a lower intake of roughage according to keepers. One of them was offered an extra portion of apples daily. The other one was given the same food as the other black rhinos housed with it, but a higher proportion of pellets, hay cobs, vegetables and fruits, and therefore less hay.

One male black rhino was known to have recurrent problems with laminitis, which resulted in a diet based on grass hay only and not on a grass-lucerne mix as the other rhinos in its institution – even though there is no logic to this practice, as high levels of protein or calcium, as present in lucerne, are not related to laminitis (Clauss & Kiefer, 2003). Additionally, it was not offered any fruits, only green leafy vegetables. It also was supplemented with vitamin C three times a week during the whole year, in contrast to the other rhinos, which received the vitamin C supplementation only seasonally during winter.

One female black rhino was offered an additional portion of horse feed based on chopped lucerne and lucerne pellets to improve its condition.

**Table S1.** Feed items and amounts used as additional extras for the rhinos on the listed occasions in facilities with white rhinos (*C. simum*) and black rhinos (*D. bicornis*)

|  |  | | White rhino | | Black rhino | |  |
| --- | --- | --- | --- | --- | --- | --- | --- |
|  |  |  | (23 facilities) | | (22 facilities) | |  |
| Feed item | Fruits | | 13 % (3) | | 59 % (13) | |  |
|  | Vegetables | | 22 % (7) | | 55 % (12) | |  |
|  | Compound feeds | | 13 % (3) | | 14 % (3) | |  |
|  | Lucerne hay | | 17 % (4) | | 9 % (2) | |  |
|  | Bread | | 13 % (3) | | 36 % (8) | |  |
|  | Trainings pellets | | 0 % (0) | | 9 % (2) | |  |
|  | Browse | | - | | 9 % (2) | |  |
|  |  | |  | |  | |  |
| Amount | | From daily amount of feed | | 22 % (5) | | 23 % (5) | |
|  | | Limited amount of extras per day/ week | | 4 % (1) | | 14 % (3) | |
|  | | Limited amount of compound feeds as extra | | 9 % (2) | | 0 % (0) | |
|  | | Extras listed on feeding plan | | 30 % (7) | | 14 % (3) | |
|  | | No extras at all | | 26 % (6) | | 0 % (0) | |
|  | | Missing information about extras | | 22 % (5) | | 50 % (11) | |
|  |  | |  | |  | |  |
| Occasion | Medical training | | 74 % (17) | | 96 % (21) | |  |
|  | Management* | | 13 % (3) | | 23 % (5) | |  |
|  | Visitor tours/ experiences | | 13 % (3) | | 32 % (7) | |  |
|  | Additionally on some days casually/ seasonally | | 4 % (1) | | 14 % (3) | |  |

***** Management refers to situations where food is used outside of training to position the animals at a barrier for examination or treatment or to relocate them to other enclosures

**Table S2.** The use of fresh grass and fresh lucerne seasonally during summer in zoos keeping white rhinos (*C. simum*) and black rhinos (*D. bicornis*)

|  |  | White rhino | Black rhino |
| --- | --- | --- | --- |
|  |  | (23 facilities) | (22 facilities) |
| Grass | seasonally | 57 % (13) | 23 % (5) |
|  | seasonally every day | 44 % (10) | 18 % (4) |
|  | seasonally instead of hay | 13 % (3) | 0 % (0) |
|  | access to grazing in some enclosures | 44 % (10) | 77 % (17) |
|  | no grass at all | 17 % (4) | 9 % (2) |
|  |  |  |  |
| Lucerne | seasonally | - | 23 % (5) |
|  | seasonally every day | - | 23 % (5) |

**Table S3.** Browse species used as feed items, browse sources and winter browse options in visited zoos keeping black rhinos (*D. bicornis*)

|  |  | Black rhino |
| --- | --- | --- |
|  |  | (22 facilities) |
| Browse species | Willow | 100 % (22) |
|  | Oak | 41 % (9) |
|  | Birch | 36 % (8) |
|  | Hazel | 27 % (6) |
|  | Hawthorne | 27 % (6) |
|  | Fruit trees | 23 % (5) |
|  | Maple | 23 % (5) |
|  | Beech | 18 % (4) |
|  | Chestnut | 18 % (4) |
|  | Rose | 14 % (3) |
|  | Conifers | 14 % (3) |
|  | Poplar | 14 % (3) |
|  | Plane tree, bramble, blackthorn, ash each | 9 % (2) |
|  | Elder, elm, bamboo, ficus, blackberry, locust, sycamore each | 5 % (1) |
|  |  |  |
| Amount of browse on average | < 5 branches/ animal/ day | 41 % (9) |
|  | 5-10 branches/ animal/ day | 23% (5) |
|  | > 10 branches/ animal/ day | 36 % (8) |
|  |  |  |
| Feeding practice | Browse lying on floor/ ground | 91 % (20) |
|  | Browse hanging | 68 % (15) |
|  | Browse placed leaning against wall/ or on items | 32 % (7) |
|  | Browse stuck into ground | 14 % (3) |
|  |  |  |
| Browse sources | Zoo gardeners | 41 % (9) |
|  | Delivered by public/ city | 41 % (9) |
|  | Cut by keepers | 36 % (8) |
|  | Own plantation | 23 % (5) |
|  |  |  |
| Winter browse option | Dried browse | 46 % (10) |
|  | Browse silage | 18 % (4) |
|  | Frozen browse | 14 % (3) |
|  | No browse for some time | 9 % (2) |
|  | Branches without leaves | 100 % (22) |
|  |  |  |
| Documentation | Browse diary | 9 % (2) |

**Table S4.** Compound feeds in zoos keeping white rhinos (*C. simum*) and black rhinos (*D. bicornis*)*

|  | White rhino | Black rhino |
| --- | --- | --- |
|  | (23 facilities) | (22 facilities) |
| Grazer pellets for zoo animals | 44 % (10) | - |
| Browser pellets for zoo animals | - | 55% (12) |
| Pellets for horses | 17 % (4) | 5 % (1) |
| Pellets for ruminants | 13 % (3) | 0 % (0) |
| Pellets for elephants | 9 % (2) | 0 % (0) |
| Pellets mixed for the zoo | 9 % (2) | 9 % (2) |
| Mineral pellets | 13 % (3) | 14 % (3) |
| Unkown | 4% (1) | 23% (5) |

***** Some institutions used more than one type of pellets

**Table S5.** Salt and other supplements fed in zoos keeping white rhinos (*C. simum*) and black rhinos (*D. bicornis*)

|  |  | White rhino | Black rhino |
| --- | --- | --- | --- |
|  |  | (23 facilities) | (22 facilities) |
| Salt | Salt lick | 35 % (8) | 36 % (8) |
|  | Salt lick with minerals | 13 % (3) | 14 % (3) |
|  | Salt (loose) | 0 % (0) | 5 % (1) |
|  |  |  |  |
| Supplements | Any kind of supplements | 44 % (10) | 59 % (13) |
|  | Vitamin E/ selenium | 13 % (3) | 27 % (6) |
|  | Vitamin C | 0 % (0) | 14 % (3) |
|  | Biotin | 9 % (2) | 18 % (4) |
|  | Multivitamin | 17 % (4) | 9 % (2) |
|  |  |  |  |
|  | Feed lime/ vitamin lime | 0 % (0) | 9 % (2) |
|  | Mineral bar for elephants | 4 % (1) | 5 % (1) |
|  | Zinc | 4 % (1) | 0 % (0) |
|  | Inulin, oak bark, carob, horse product (liver & kidney protection), horse product (anabolic vitamin mix), horse product (psyllium husks for sand expulsion), horse product (gastric protection) each | 0 % (0) | 5 % (1) |
|  |  |  |  |
| Supplements for single animals | Horse product (liver & kidney protection) | 0 % (0) | 5 % (1) |
|  | Horse product (joint protection) | 0 % (0) | 5 % (1) |
|  | Carotene bar | 4 % (1) | 0 % (0) |
|  | Horse product (for breeding horses) | 4 % (1) | 0 % (0) |

**Table S6.** Frequency of feeding and feeding practices and locations in facilities with white rhinos (*C. simum*) and black rhinos (*D. bicornis*).

|  |  | White rhino | Black rhino |
| --- | --- | --- | --- |
|  |  | (23 facilities) | (22 facilities) |
| Feedings frequency | 2 feedings | 91 % (21) | 86 % (19) |
| per day | 3 or more feedings | 9 % (2) | 14 % (3) |
|  | Seasonally only 1 feeding | 13 % (3) | 5 % (1) |
|  |  |  |  |
| Location | Inside feeding | 100 % (23) | 100 % (22) |
|  | Outside feeding | 100 % (23) | 77 % (17) |
|  |  |  |  |
| Inside feeding | 1 fixed feeding spot | 78 % (18) | 82 % (18) |
|  | Various feeding spots | 22 % (5) | 18 % (4) |
|  | Single females share feed sometimes | 74 % (17) | 0 % (0) |
|  |  |  |  |
| Outside feeding | 1 feeding spot | 9 % (2) | 27 % (6) |
|  | 2-3 feeding spots | 46 % (10) | 57 % (13) |
|  | 4 or more feeding spots | 14 % (3) | 35 % (8) |
|  | Constant location of feeding spots | 74 % (17) | 77 % (17) |
|  | Group feeding | 100 % (23) | 27 % (6) |
|  | Some feed possibly shared with other species in mixed exhibits | 52 % (12) | 18 % (4) |
|  |  |  |  |
| Hay placement | Hayracks | 13 % (3) | 41 % (9) |
|  | Hay on the floor/ ground | 100% (23) | 91 % (20) |
|  | Concrete/ stone spot for hay | 26 % (6) | 14 % (3) |
|  | Other place for hay* | 4 % (1) | 23 % (5) |

***** Placed on top or in items, hay nets, feeding automat

**Table S7.** Nutritional components on average in the diet of visited institutions with white rhinos (*C. simum*) and black rhinos (*D. bicornis*).

|  | White rhino |  |  | Black rhino |  |  |
| --- | --- | --- | --- | --- | --- | --- |
|  | (22 facilities) |  |  | (22 facilities) |  |  |
|  | Mean (±SD) | Min | Max | Mean (±SD) | Min | Max |
| Total non-forage kg DM | 1.42 (±1.43) | 0 | 3.60 | 2.87 (±3.54) | 0.45 | 9.77 |
| Ratio non-forage: forage | 0.08 (±0.10) | 0 | 0.25 | 0.34 (±0.87) | 0.03 | 1.97 |
| Assumed percentage forage | 93 (±8) | 80 | 100 | 79 (±24) | 34 | 97 |
| Ratio compound feeds: non-forage | 0.81 (±0) | 0 | 1.00 | 0.81 (±0.06) | 0.25 | 1.00 |
|  |  |  |  |  |  |  |
| Compound feeds kg DM | 1.28 (±1.43) | 0 | 3.60 | 2.34 (±3.62) | 0.45 | 9.59 |
| Single feeds kg DM | 0.12 (±0) | 0 | 1.24 | 0.15 (±0) | 0 | 0.90 |
| Vegetables kg DM | 0.01 (±0) | 0 | 0.32 | 0.31 (0.06) | 0 | 1.50 |
| Fruits kg DM | 0 (±0) | 0 | 0.03 | 0.07 (±0.02) | 0 | 0.45 |
|  |  |  |  |  |  |  |
| Compound feeds g/kg^0.75^ | 4.71 (±5.25) | 0 | 13.71 | 12.05 (±17.13) | 2.21 | 45.60 |
| Single feeds g/kg^0.75^ | 0.41 (±0) | 0 | 4.20 | 0.77 (±0) | 0 | 4.54 |
| Vegetables g/kg^0.75^ | 0.05 (±0) | 0 | 1.11 | 1.57 (±0.30) | 0 | 8.05 |
| Fruits g/kg^0.75^ | 0 (±0) | 0 | 0.10 | 0.35 (±0.11) | 0 | 2.15 |

**Table S8.** Average values of fecal score and colour in facilities with white rhinos (*C. simum*) and black rhinos (*D. bicornis*).

|  |  | White rhino | Black rhino |
| --- | --- | --- | --- |
|  |  | (23 facilities) | (22 facilities) |
| Score | 1 | 0 % (0) | 5 % (1) |
|  | 2 | 91 % (21) | 32 % (7) |
|  | 3 | 4 % (1) | 59 % (13) |
|  | 4 | 0 % (0) | 5 % (1) |
|  | Not assessable | 4 % (1) | 0 % (0) |
|  |  |  |  |
| Colour | Light brown | 0 % (0) | 0 % (0) |
|  | Medium brown | 91 % (21) | 73 % (16) |
|  | Dark brown | 9 % (2) | 27 % (6) |

Clauss, M., & Kiefer, B. (2003). Digestive acidosis in captive wild herbivores – implications for hoof health. *Verhandlungsbericht Erkrankungen der Zootiere, 41*, 57-70.
